# Efficiency of atorvastatin on in-hospital mortality of patients with acute aortic dissection (AAD): study protocol for a randomized, open-label, superiority clinical trial

**DOI:** 10.1186/s13063-021-05237-1

**Published:** 2021-04-14

**Authors:** Yequn Chen, Nianling Xiong, Xin Wang, Shiwan Wu, Liangli Hong, Xiru Huang, Chang Chen, Weiping Li, Bin Wang, Shu Ye, Xuerui Tan

**Affiliations:** 1 The First Affiliated Hospital of Shantou University Medical College, Shantou, 515041 Guangdong China; 2 Clinical Research Center, The First Affiliated Hospital of Shantou University Medical College (SUMC), Shantou, China; 3 Shantou University Medical College, Shantou, 515041 Guangdong China; 4 Clinical Cohort Research Center, The First Affiliated Hospital of Shantou University Medical College, Shantou, China; 5 Department of Cardiovascular Sciences and NIHR Leicester Biomedical Research Centre, University of Leicester, Leicester, UK

**Keywords:** Acute aortic dissection, Atorvastatin, Statins, In-hospital mortality

## Abstract

**Background:**

Dyslipidemia and local inflammation at sites of lipid deposition on blood vessel walls have been demonstrated to be risk factors for patients with acute aortic dissection (AAD). Statins have anti-inflammatory and lipid-lowering effects, which suggest that statins may play an important role in the prevention and treatment of AAD. Some retrospective studies show that statins can protect patients with aortic dissection. However, the effect of statins on the survival of AAD patients has been scarcely investigated, especially in randomized trials. In this study, we will perform a randomized clinical trial to understand whether statins can reduce in-hospital mortality of AAD patients.

**Methods:**

A total of 384 subjects diagnosed with AAD in the First Affiliated Hospital of Shantou University Medical College will be recruited. Participants will be randomly divided into an atorvastatin-treated or control group. The primary outcome will be the in-hospital mortality at 30 days.

**Discussion:**

This study is designed to verify the efficacy of atorvastatin on reducing in-hospital mortality of patients with AAD. The aim is to provide a new means of improving survival as a complement to conventional drug therapy.

**Trial registration:**

Chinese Clinical Trials Registry ChiCTR1900023515. Registered on 1 June 2019.

## Background

Acute aortic dissection (AAD) is relatively uncommon but is considered a life-threatening, potentially fatal condition [1]. A recent International Registry of Acute Aortic Dissection (IRAD) study shows that 87–90% of AAD is treated surgically, and drug treatment only accounts for 7–8%. Nevertheless, the IRAD study indicates that in-hospital surgical mortality for AAD remains approximately 20% [2], indicating improvement of the long-term outcome of patients with aortic diseases requires further optimization [3]. However, in addition to strict control of blood pressure and heart rate, the effect of other drugs on AAD is still controversial and lacking strong clinical evidence.

Dyslipidemia and atherosclerosis are important risk factors for AAD [4]. Lipid deposition in the vascular wall can lead to local inflammation [5] and promote the release of inflammatory mediators that destroy the vascular wall, leading to aortic dissection (AD) [6]. Statins are classical drugs for lowering low-density lipoprotein cholesterol (LDL-C) levels, and are both anti-inflammatory and anti-atherosclerotic [7], suggesting that statins may delay progression of AAD.

Recent reports have shown that statins have anti-inflammatory effects and can decrease morbidity and mortality following surgical repair of aneurysms in patients with abdominal aortic aneurysms and aneurysm expansion [8–11], and several experimental studies demonstrate the protective effect of statins [12–14]. A retrospective study showed that patients with AAD who used statins had a significantly better prognosis than those who did not [15]. Also, a prospective and randomized comparative study of patients with uncomplicated acute type B aortic dissection (ABAD) showed that pitavastatin treatment has a suppressive effect on aortic arch dilatation [3]. However, a retrospective review points out that statins do not improve in-hospital and long-term outcomes in patients with Stanford B aortic dissection treated with endovascular aortic repair [16]. Although these studies have explored the effects of statins on aortic disease [3, 14–17], the results remain controversial. The aim of this randomized clinical trial study is to understand the efficiency of atorvastatin on in-hospital mortality of patients with AAD. We propose that AAD patients treated with statins will have lower in-hospital mortality than patients without.

## Methods

### Study design

This prospective trial was designed as a single-center randomized, open-label, superiority clinical trial with patients allocated 1:1, to be conducted at the First Affiliated Hospital of Shantou University Medical College, Shantou, Guangdong, China. The protocol was designed according to the Standard Protocol Items: Recommendations for Interventional Trials (SPIRIT) 2013 Statement.

### Study objectives and hypothesis

The primary objective of this trial is to assess the effect of statins on in-hospital mortality of AAD patients. The secondary objective is to determine the incidence of liver dysfunction (alanine aminotransferase (ALT)/aspartate aminotransferase (AST) > 3 times the normal value). We predict that AAD patients treated with statins will have lower in-hospital mortality than patients without.

### Ethics issues

The present study protocol was approved by the Committee of Ethics of the First Affiliated Hospital of Shantou University Medical College (Number: 2019038).

### Participant recruitment

For the clinical study registered on 1 June 2019, participants will be screened after registration and will be diagnosed for AAD by means of computed tomography angiography (CTA) [18] in The First Affiliated Hospital of Shantou University Medical College. Patients who may qualify for the study will be referred by an emergency physician, community hospital doctor or member of a healthcare hospital research team. A research team consists of clinicians who are familiar with AAD diagnosis and treatment. In addition, the researchers will receive appropriate training regarding all the relevant information pertaining to the study. Those who are willing to take part in the study will be carefully evaluated according to the inclusion and exclusion criteria.

### Criteria

Inclusion criteria:

1. Patient agrees to cooperate with all study procedures;
2. Age > 20 years old;
3. Physical status permits oral medication;
4. Cholesterol < 5.1 mmol/l, triglyceride < 1.7 mmol/l;
5. Provision of written informed consent.

Exclusion criteria:

1. Liver dysfunction: alanine aminotransferase (ALT)/aspartate aminotransferase (AST) > 3 times the normal value;
2. Breast-feeding or pregnancy (for women of child-bearing age, the human chorionic gonadotropin level will also be measured before drug administration);
3. Previous history of coronary atherosclerotic heart disease;
4. Statins used within 6 months before inclusion;
5. Participation in another clinical trial within 6 months;
6. Allergy to statins.

### Informed consent

Prior to enrollment, trained researchers will introduce the objective and main aspects of the trial to the patients. Patients will also receive information sheets and then be able to have an informed discussion with the participating consultant. Patients will also be informed of the probable benefits and potential risks and assured that participation is entirely voluntary. Patients who meet all of the inclusion criteria and none of the exclusion criteria will be enrolled after providing written informed consent. The personal information of all participants will always be kept confidential.

### Randomization and allocation

**Fig. 1:**
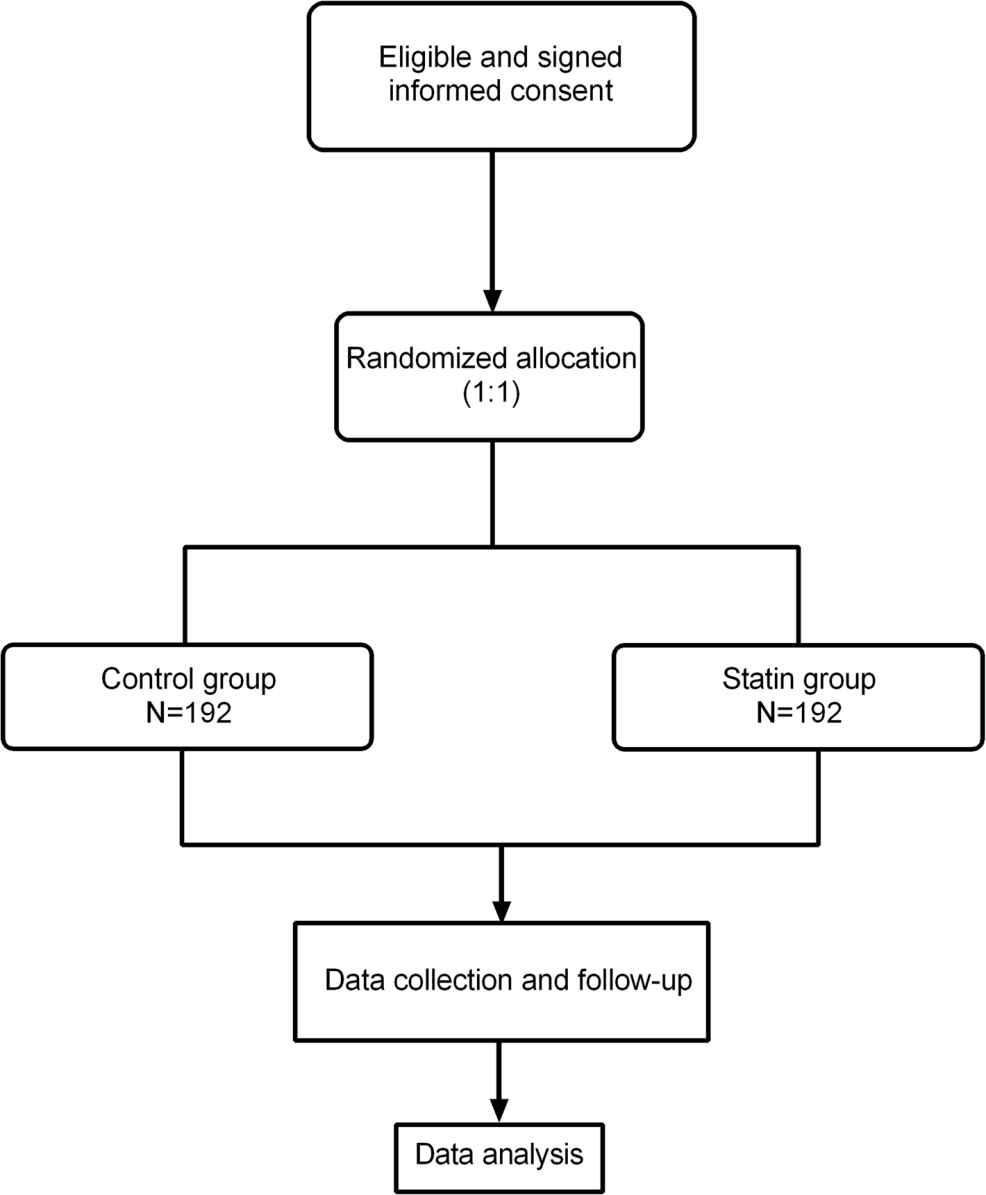
Subject distribution among groups in the study

According to the order of the participants enrolled, they will be randomly assigned to the control or statin group by using a corresponding random number and envelope method for allocation concealment (patients will be assigned to a corresponding group according to the allocation sequence in an envelope that will be distributed to the research implementation personnel after the allocation sequences are packed in an opaque envelope by separate staff). Balance in allocation across the study participants will be enhanced through stratification by the type of AAD (two types: type A, type B). To avoid the imbalance caused by simple randomization, we introduce block randomization. The block length was 4 with two groups in this study (statins group and control group). The allocation sequence will be generated at a 1:1 (statin/control) ratio using the random number generator in the SPSS statistical software package (SPSS Inc., Chicago, IL, USA, version 23) (Fig. 1).

### Blinding

This is an open-label trial. Trial participants, investigators, care givers, outcome assessors, and date analysts are not blinded to group assignment.

### Interventions

Participants will be randomized into two equal groups: statin (atorvastatin) or control group as soon as clinical conditions permit. The statin group will be required to take atorvastatin 20 mg daily (known to be a safe dose effective in lipid-lowering and anti-inflammatory activity [19–21]), and the control group will be required to not take statins during study period. Atorvastatin will be provided by Pfizer (New York, USA).

**Fig. 2:**
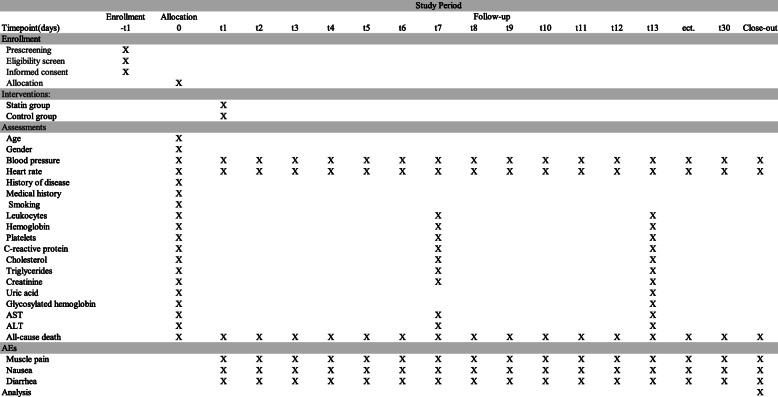
Schedule of enrollment, interventions and assessments for the trial

The scheduled duration of treatment is the duration of the hospital stay unless a serious adverse event occurs or the participant quits. The detailed study schedule is listed in Fig. 2.

### Data handling and record keeping

Once the subjects are formally enrolled, case report forms (CRFs) will be established. The CRF will be used to record data for all participants. This task will be completed by the researchers, who will also enter the data into an electronic database, and CRFs will be double entered.

### Follow-up

Patients will be followed up daily up to 30 days post randomization unless death or discharge occurs. The average length of stay of patients with AAD from 2013 to 2018 was 13 days in the First Affiliated Hospital of Shantou University Medical College, so the expected time window may be 2 weeks. Outcome will be collected at the time of the participant’s death, discharge from the hospital or at the 30th day. Each subject’s survival status (surviving/deceased), blood pressure, heart rate, and AEs will be recorded on the CRF after each conversation. At the 7th and 13th day, we will also measure leukocytes, hemoglobin, platelets, C-reactive protein, cholesterol, triglycerides, creatinine, uric acid, glycosylated hemoglobin, AST, and ALT. Face-to-face adherence reminder sessions will take place at the initial product dispensing and each study visit thereafter.

### Withdrawal

Patients may be withdrawn from the study for any of the following reasons:

1. They may choose to withdraw for any reason;
2. They have abnormalities in ALT/AST (> 3 times upper limit of normal) after drug administration;
3. The patient wants to accept other kinds of statins;
4. Other situations: based on the investigator’s discretion, the patient is no longer eligible for the study for any reason.

### Sample size

Preliminary data indicates that acute aortic dissection is associated with an in-hospital mortality of 27.4% [18]. We predict that treatment with atorvastatin will reduce mortality to 12.4%, while no change will be seen in the control group, which will show a mortality of 27.4%. To obtain 90% power to demonstrate a statistically significant difference (two-sided test, alpha = 0.05) between the two treatments, we anticipate that 307 participants will need to be recruited with154 for the control group and 153 for the statin group. This corresponds to a hazard ratio of 0.4135. Given the possibility of exclusion or lost follow-ups, an increase of 20% will be required for the sample size, bringing the sample size to 192 per arm for a total of 384 participants to be enrolled. All calculations are done through PASS software (NCSS, America, version 11), using the log-rank test, Freedman method.

### Data collection and management

At enrollment, we will collect the demographic information of the participants, including age, gender, blood pressure, heart rate, previous disease history, medical history, smoking history. Leukocytes, hemoglobin, platelets, C-reactive protein, cholesterol, triglycerides, creatinine, uric acid, glycosylated hemoglobin, AST, and ALT will be measured at baseline, the 7th, and 13th day. Here, 2 mL fasting venous blood will be taken for measurement in the morning. All these blood parameters will be collected. At discharge or death of the patient, data will be collected on patient status, medication in the hospital, diagnosis and comorbidity. All data will be transferred to a database by duplicate entry using EpiData software (Denmark, version 3.1), and will be stored in the scientific research platform of the First Affiliated Hospital of Shantou University Medical College, which is a secure server with limited access and supported for anonymity, analysis and review.

### Outcome measurements

The primary outcome of this study will be the in-hospital mortality at 30 days. We will calculate in-hospital mortality by counting all-cause death. Secondary outcome will be the incidence of liver dysfunction, as measured through ALT and AST at the 7th and 13th day. The expected trial target is fewer deaths in the statin group compared to the control group.

### Statistical and analytical plans

A comprehensive statistical analysis plan will be prepared before database locking. All analyses will be conducted according to the intention-to-treat (ITT) principles. The primary outcome is binary, it will be expressed as frequency and percentage, a chi-square test will be used to analyze. All statistical analyses will be two-sided, and statistical significance will be set at 0.05. Continuous data such as blood pressure, heart rate and blood parameters, will be presented as mean ± SD; Categorical data, such as previous disease history and smoking history, will be expressed as frequency and percentage of patients in each category. The Kolmogorov-Smirnov test will be used to confirm the normality of the distribution of continuous data, if *P* < 0.05 indicates the data is not normally distributed, advanced raw data conversion (such as logarithmic conversion) will be performed to see whether the data conform to a normal distribution. If not, the rank-sum test will be selected. Otherwise, Student’s *t* tests will be used for continuous data. The chi-square test will be used for categorical data. Survival curves will be determined by the Kaplan-Meier method for survival analysis. The difference in changes between the groups will be analyzed using both Student’s *t* test and analysis of covariance to adjust for the baseline values. A *p* value of < 0.05 will be used to indicate statistical significance. The missing data will be handled by using Mean/Mode Completer. If the missing data is numerical, it will be filled according to the average value of all other objects; if the missing data is non-numerical, then it will be supplemented by the value with the most frequent value of all other objects according to the mode principle in statistics. SPSS statistical software (SPSS Inc., Chicago, IL, USA, version 23) will be used for statistical analysis.

### Monitoring

This study will be performed in accordance with the approved protocol. The data and safety monitoring committee of the First Affiliated Hospital of Shantou University Medical College will review and interpret the data generated from the study in order to ensure the safety of the participants and the integrity of the research data. The committee consists of five independent researchers who are independent of the funders and have no conflict of interest with this study. The data and safety monitoring committee will review the data after 25%, 50%, and 75% enrollment to monitor the study progress and all adverse events (AEs) that may occur. Computer-generated and time-stamped audit trials will also be implemented for tracking changes in the electronic source documentation to ensure the integrity of the research data.

### Auditing

An audit by the ethics committee’s representatives during the course of the study will be performed according to national regulations. All protocol modifications will be communicated to the relevant parties.

### Safety and adverse events

The most common AEs related to the intake of atorvastatin are expected to be liver enzyme abnormalities, muscle pain, nausea, and diarrhea. Participants showing any adverse event will be treated appropriately by doctors, and the project will cover the cost of adverse events. Adverse reactions will be checked at every visit. For any AE that occurs, all details, including the time of occurrence, symptoms and signs, degree, duration, laboratory findings, treatment, outcomes, and causal relationship with the treatment, will be recorded in the CRF. Serious AEs will be reported to the Research Ethics Committee within 24 h, which will decide whether any additional measures should be taken. The total number of patients with AEs related to the study drug (certain, probable, possible) and the total number of patients with AEs leading to discontinuation of study treatment will be summarized.

### Amendment

All proposed protocol changes will be recorded in a protocol amendment and this will be submitted to the Committee of Ethics and the regulatory authority for approval.

### Limitations

The limitations of this study should also be considered, including confirming that patients can diet normally before being formally included in the study, which may exclude critically ill patients whose deaths would occur within several hours of onset and would not be reported, thereby reducing the mortality rate reported for AAD. For surgical patients, preoperative fasting is often required, and patients in this group often need to wait until a normal diet is restored after surgery before they can be included in the study, which may also exclude critically ill patients whose deaths would occur before normal dieting is restored and would not be reported, thereby reducing the mortality rate reported for AAD. Considering the safety of atorvastatin, a low dose of 20 mg/day will be used in this study, and any possible effects may not be very significant. This is an exploratory study. If there is no significant positive effect at the end, but the results suggest it is safe and does not increase mortality, effectiveness may be observed by subsequently increasing the dose. We will explore the most appropriate dose in the next study. As atorvastatin is a tablet drug, we have sought several companies including Pfizer for suitable tablet placebo, while we failed to find out such placebo that are totally the same in color, shape, smell, and taste. So this is an open-label trial which may weaken its persuasiveness. However, if the results of this study are positive, it will provide evidence in reducing in-hospital mortality in patients with AAD, we will continue to carry out multicenter double-blind study to verify the results of this experiment.

## Discussion

AAD confers poor prognosis if left unmanaged [22]. Currently, although surgical treatment can significantly reduce the mortality, the mortality remains high, which indicates other interventions are needed to further reduce the mortality rate. The 17-year trends in the presentation, diagnosis, and hospital outcomes of AAD from the IRAD were retrospectively reviewed by Linda et al. [2], who showed that besides angiotensin receptor blockers and beta-blockers used to treat AAD, statins are being used with increasing frequency.

Numerous studies indicate that statins have multiple beneficial actions on the cardiovascular system through improvement of endothelial dysfunction, inflammation, oxidative stress, and stabilization of atherosclerotic plaques [23]. Endothelial dysfunction has been recognized as an independent predictor of cardiovascular disease, and statins significantly ameliorate endothelial dysfunction [24, 25]. The reason why we choose atorvastatin is that atorvastatin is the most widely used statin and has been administered in a variety of settings, the incidence of AEs with atorvastatin 10–40 mg in patients of Asian origin is low, and compared with other statins, atorvastatin is safer and has greater beneficial effects on oxidative stress and endothelial function [26–28].

The objective of this trial is to assess the effect of statins on the mortality of AAD patients. If the results of our investigation are positive, this study will provide evidence regarding the value of statins as an intervention to decrease in-hospital mortality in patients with AAD. Also, if there is no significant positive effect at the end, but the results suggest it is safe and does not increase mortality, effectiveness may be observed by subsequently increasing the dose. We will explore the most appropriate dose in the next study.

## Trial status

The version of this protocol is 2.0 (date 20201225). It is currently recruiting patients.

## Data Availability

The project owner will have access to the final trial dataset. The trial data will be made public and manuscript(s) will be published in a peer review clinical journal regardless of whether the results of the trial are positive, negative or inconclusive.

## Abbreviations

AAD: Acute aortic dissection
ABAD: Acute type B aortic dissection
AD: Aortic dissection
ALT: Alanine aminotransferase
AST: Aspartate aminotransferase
CONSORT: Consolidated Standards of Reporting Trials
CRF: Case report forms
CTA: Computed tomography angiography
IRAD: International Registry of Acute Aortic Dissection
IRB: Institutional review board
ITT: Intention-to-treat
LDL-C: Low-density lipoprotein cholesterol
RCT: Randomized controlled trial
TEAE: Treatment-emergent adverse events
